# Novel dynamics and Cooper-pair momentum measurement in Fulde-Ferrell-Larkin-Ovchinnikov superfluids on optical lattices

**DOI:** 10.1016/j.isci.2026.116065

**Published:** 2026-05-26

**Authors:** Shuning Tan, Jiayi Shi, Peng Zou, Tianxing Ma, Huaisong Zhao

**Affiliations:** 1 Key Laboratory for Microstructural Material Physics of Hebei Province, School of Science, Yanshan University, Qinhuangdao 066004, P.R. China; 2 Centre for Theoretical and Computational Physics, College of Physics, Qingdao University, Qingdao 266071, P.R. China; 3 School of Physics and Astronomy, Beijing Normal University, Beijing 100875, P.R. China

**Keywords:** physics, condensed matter physics, applied sciences

## Abstract

Determining the center-of-mass (COM) momentum of Cooper pairs in unconventional superconductors or superfluids is a topic of great interest in condensed matter physics and ultracold atomic gases. Theoretically, we investigate the dynamical excitations of a two-dimensional spin-polarized attractive Hubbard model on a square optical lattice under an effective Zeeman field by computing the density and spin dynamical structure factors, focusing on phase transition from a Bardeen-Cooper-Schrieffer (BCS) superfluid to a Fulde-Ferrell-Larkin-Ovchinnikov (FFLO) superfluid. In the FFLO superfluid, besides the phonon mode in the density channel, a low-energy bogolon mode emerges in the spin channel, which is associated with Bogoliubov quasiparticles on a Bogoliubov Fermi surface. Moreover, the dynamical excitations exhibit pronounced anisotropy in momentum space due to the finite COM momentum. At half filling, the roton mode around [*π*, *π*] evolves from a point-like minimum into a ring structure shifted by the COM momentum across the BCS-FFLO transition, providing a roton-based protocol to extract the COM momentum. These predictions provide key insights for confirming the existence of FFLO superfluids and understanding their dynamical excitation spectra.

## Highlights

• We study the anisotropic dynamic excitations of an FFLO superfluid on optical lattices
• The roton mode provides an effective way to determine the center-of-mass momentum
• The phonon mode in an FFLO state exhibits a period of π in angle
• A bogolon mode emerges in the spin channel and its speed is larger than the sound speed

## Graphical abstract

**Figure:**
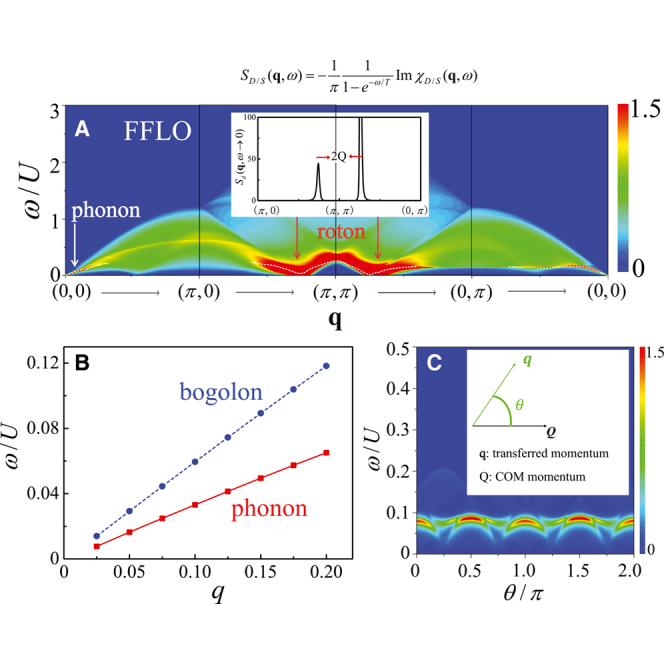

## Introduction

Unlike conventional Bardeen-Cooper-Schrieffer (BCS) superconductors, where Cooper pairs carry zero center-of-mass (COM) momentum **Q** = 0, there exists a class of exotic superconductors with finite COM momentum, such as the pair-density wave (PDW) superconductors and Fulde-Ferrell-Larkin-Ovchinnikov (FFLO) superconductors. The PDW state arises without an applied Zeeman field while the Fulde-Ferrell-Larkin-Ovchinnikov (FFLO) state is stabilized under an applied Zeeman field. Since the experimental realization of the PDW in various superconductors,^1^^,^^2^^,^^3^^,^^4^^,^^5^^,^^6^^,^^7^^,^^8^^,^^9^ the physical properties of PDW system have attracted significant attention.^10^^,^^11^ Meanwhile, the FFLO state have been discussed in the multiband iron-based superconductors.^8^^,^^12^^,^^13^^,^^14^ As highly tunable and clean systems, the ultracold atomic gases provide an excellent platform for simulating such novel many-body physics in condensed matter physics.^15^^,^^16^ In particular, the FFLO superfluid state are widely expected to exist in polarized Fermi gases,^17^^,^^18^^,^^19^^,^^20^^,^^21^^,^^22^^,^^23^^,^^24^^,^^25^^,^^26^ motivating extensive experimental and theoretical efforts to realize it in ultracold atomic gases.^26^^,^^27^^,^^28^^,^^29^^,^^30^^,^^31^^,^^32^^,^^33^^,^^34^ Understanding how the characteristic physical quantities evolve across the BCS-FFLO transition becomes a key issue. Our previous studies exhibited some significant differences on dynamical excitations between a conventional BCS superfluid and various exotic superfluid phases, including topological superfluid,^35^^,^^36^^,^^37^ Sarma phase,^38^ and one-dimensional FFLO gases.^39^ Recent progress shows that the collective mode can distinguish PDW and charge-density-wave (CDW) states.^40^ These studies indicate that dynamical excitations can serve as a sensitive tool for identifying the FFLO superfluids and discriminating them from the conventional BCS superfluid.

In conventional BCS superfluids, the Fermi surface disappears due to the opening of the pairing gap, so that the single-particle excitations are gapped and appear only above a finite threshold energy. Within this energy range, the single-particle excitations and collective modes can be clearly separated. As a result, the collective phonon mode can be measured distinctly. However, in the FFLO superfluids, the Fermi surface persists, and the quasiparticle spectrum remain gapless and form a Bogoliubov Fermi surface (BG-FS),^41^^,^^42^^,^^43^^,^^44^^,^^45^^,^^46^ where the low-energy quasiparticle excitations near the Fermi energy are described by the Bogoliubov quasiparticles (bogolons). This Bogoliubov Fermi liquid exhibits distinct physical properties, such as the odd-frequency pairing,^44^^,^^47^^,^^48^ the unconventional impurity effect on the single-particle spectra and density of states (DOSs),^46^ a Drude-like optical conductivity contrasting with that of d-wave superconductors.^49^ Therefore, in addition to the conventional single-particle excitations, the low-energy dynamical excitations are composed of the phonon and bogolon. Moreover, in the FFLO superfluids, the single-particle excitations strongly compete with these collective modes. Hence, this competition makes it essential to simultaneously calculate the full dynamical excitations. These dynamical excitations can be probed through the dynamical structure factors, which are two-body correlation observables.^50^^,^^51^^,^^52^ In the ultracold atomic gases, the dynamical structure factors can be directly measured using two-photon Bragg spectroscopy^53^^,^^54^^,^^55^^,^^56^^,^^57^^,^^58^^,^^59^^,^^60^ and can also be simulated through various numerical approaches.^61^^,^^62^^,^^63^^,^^64^

Optical lattices have become a widely used platform for simulating the physical models in condensed matter physics, such as the Fermi-Hubbard model.^65^^,^^66^^,^^67^^,^^68^^,^^69^^,^^70^^,^^71^^,^^72^^,^^73^^,^^74^^,^^75^ The attractive Fermi-Hubbard model is strongly associated with the superfluid phenomenon and has been extensively studied through numerous experiments^76^^,^^77^^,^^78^^,^^79^^,^^80^ and theoretical studies.^81^^,^^82^^,^^83^^,^^84^^,^^85^^,^^86^ Compared with the continuous gases characterized by an extremely narrow parameter window, the FFLO states on an optical lattice exhibit significantly broader parameter tunability,^24^ thereby enhancing the experimental feasibility. The collective modes of the lattice FFLO superfluid have been investigated through several numerical simulations,^87^^,^^88^^,^^89^^,^^90^^,^^91^ revealing that the collective modes exhibits the anisotropic behavior. For instance, the sound speed parallel to **Q** is greater than that perpendicular to **Q**,^89^ and two asymmetric roton-like collective modes have been identified.^90^ Nevertheless, until now, a systematic investigation of the full dynamical excitations is still lacking, particularly concerning the phonon, bogolon, roton modes, as well as their competition with the single-particle excitation continua. In particular, an experimental protocol to extract the COM momentum **Q** from the dynamical excitations remains an open question.

In this paper, we theoretically compute the density and spin dynamical structure factors of a two-dimensional (2D) spin-polarized attractive Fermi-Hubbard model on a square lattice and investigate the evolution of the full dynamical excitations across the BCS-FFLO transition within the random phase approximation (RPA).^92^^,^^93^^,^^94^^,^^95^ A central result is a roton-based measurement protocol for the Cooper-pair momentum. At half filling, the roton mode near [*π*, *π*] in density dynamical structure factor enables a direct extraction of the COM momentum magnitude from the roton minimum. In ultracold atomic gases, the response theories based on RPA have been demonstrated to describe the dynamical excitations reliably. For three-dimensional (3D) Fermi superfluids, they even yield quantitatively consistent results when compared with a two-photon Bragg scattering spectra,^57^^,^^96^^,^^97^ and in 2D system they reproduce qualitative results found in quantum Monte Carlo calculations.^52^^,^^61^^,^^98^ We therefore expect that our theoretical strategy provides a qualitatively accurate prediction for a 2D lattice system in the intermediate-coupling regimes.^99^

This paper is organized as follows. We first derive the mean-field Green’s functions of a 2D spin-polarized attractive Fermi-Hubbard model on a square lattice using the equations of motion approach. We then formulate the RPA response functions and obtain the dynamical structure factors. Next, we present and discuss the dynamical structure factors at half-filling, focusing on anisotropic collective modes and complex single-particle excitations. We further analyze the doping dependence of the dynamical excitations. Finally, we discuss the physical implications and summarize our conclusions. Additional calculation details are provided in the supplemental information.

## Results

### Model and Hamiltonian

For a polarized two-component 2D attractive Fermi-Hubbard model on an optical lattice, an FFLO superfluid state may exist under proper interaction strength and external Zeeman field. Consequently, this system becomes an effective platform to investigate the physical properties of the FFLO superfluids. Its Hamiltonian in momentum space is given by:

(Equation 1) $$H=\underset{k,\sigma }{\sum }\xi_{k\sigma }C_{k\sigma }^{†}C_{k\sigma }-U\underset{k}{\sum }C_{k↑}^{†}C_{\text{Q}-\text{k}↓}^{†}C_{\text{Q}-\text{k}↓}C_{k↑},$$

where *ξ*_**k***σ*_ = *ϵ*_**k**_ − *hσ*_*z*_, *ϵ*_**k**_ = −*Ztγ*_**k**_ − *μ*, $\gamma_{k}=\left(\cos k_{x}+\cos k_{y}\right)/2$, and *σ*_*z*_ = 1(−1) for spin *σ* = *↑* (*↓*). Here, *μ* is the average chemical potential and *h* is the effective Zeeman field. For a 2D square lattice, the coordination number is *Z* = 4. *C*_**k***σ*_ and $C_{k\sigma }^{†}$ denote the annihilation and creation operators for a fermion with momentum **k** and spin *σ* = *↑*, *↓*. The vector **Q** denotes the COM momentum. The on-site attractive interaction between the opposite-spin atoms is characterized by the Hubbard energy *U* > 0. In the following discussions, we take *U* as the unit of energy and the lattice constant *a*_0_ as the unit of length.

In an FF superfluid state, the pairing gap (the order parameter) is defined as, $\Delta^{*}=U<C_{k↑}^{†}C_{\text{Q}-\text{k}↓}^{†}>$, and take the plane wave form, Δ(**r**) = Δ*e*^*i***Q**⋅**r**^. Within the mean-field approximation, the four-operator interaction term is decoupled into a two-operator form: $UC_{k↑}^{†}C_{\text{Q}-\text{k}↓}^{†}C_{\text{Q}-\text{k}↓}C_{k↑}=\Delta^{*}C_{\text{Q}-\text{k}↓}C_{k↑}+\Delta C_{k↑}^{†}C_{\text{Q}-\text{k}↓}^{†}-\Delta^{2}/U$. Consequently, the mean-field Hamiltonian is obtained as, $H_{MF}=\sum_{k,\sigma }\xi_{k\sigma }C_{k\sigma }^{†}C_{k\sigma }+\frac{\Delta^{2}}{U}-\sum_{k}\left(\Delta C_{k↑}^{†}C_{\text{Q}-\text{k}↓}^{†}+\Delta^{*}C_{\text{Q}-\text{k}↓}C_{k↑}\right)$. We define the spin-up (spin-down) particle number density Green’s function, $G_{↑}\left(k,\tau -\tau^{\prime }\right)=-\left\langle T_{\tau }C_{k↑}\left(\tau \right)C_{k↑}^{†}\left(\tau^{\prime }\right)\right\rangle$ $\left(G_{↓}\left(k,\tau -\tau^{\prime }\right)=-\left\langle T_{\tau }C_{k↓}\left(\tau \right)C_{k↓}^{†}\left(\tau^{\prime }\right)\right\rangle \right)$, and the singlet pairing one $\Gamma^{†}\left(k,\tau -\tau^{\prime }\right)=-\left\langle T_{\tau }C_{\text{Q}-\text{k}↓}^{†}\left(\tau \right)C_{k↑}^{†}\left(\tau^{\prime }\right)\right\rangle$, respectively. Due to the Zeeman field, *G*_*↑*_ ≠ *G*_*↓*_. Using the equations of motion for these Green’s functions, these three Green’s functions are solved as:

(Equation 2a) $$\begin{array}{ll} G_{↑}\left(k,\omega \right) & =\frac{U_{k}^{2}}{\omega -E_{k}^{\left(1\right)}}+\frac{V_{k}^{2}}{\omega +E_{k}^{\left(2\right)}} \end{array}$$

(Equation 2b) $$\begin{array}{ll} G_{↓}\left(k,\omega \right) & =\frac{U_{k}^{2}}{\omega -E_{\text{Q}-\text{k}}^{\left(2\right)}}+\frac{V_{k}^{2}}{\omega +E_{\text{Q}-\text{k}}^{\left(1\right)}} \end{array}$$

(Equation 2c) $$\begin{array}{ll} \Gamma^{†}\left(k,\omega \right) & =\frac{\Delta^{*}}{2E_{k}}\left(\frac{1}{\omega -E_{k}^{\left(1\right)}}-\frac{1}{\omega +E_{k}^{\left(2\right)}}\right), \end{array}$$

where $U_{k}^{2}=0.5\left[1+0.5\left(\xi_{k}+\xi_{\text{Q}-\text{k}}\right)/E_{k}\right]$, $V_{k}^{2}=0.5\left[1-0.5\left(\xi_{k}+\xi_{\text{Q}-\text{k}}\right)/E_{k}\right]$. The quasiparticle spectra $E_{k}^{\left(1\right)}=E_{k}+\left(\xi_{k}-\xi_{\text{Q}-\text{k}}\right)/2-h$, $E_{k}^{\left(2\right)}=E_{k}-\left(\xi_{k}-\xi_{\text{Q}-\text{k}}\right)/2+h$, where $E_{k}=\sqrt{{\left(\xi_{k}+\xi_{\text{Q}-\text{k}}\right)}^{2}/4+\Delta^{2}}$. Furthermore, the dispersion of $-E_{k}^{\left(2\right)}$ always lies below the Fermi energy, while $E_{k}^{\left(1\right)}$ crosses the Fermi energy.

Based on the mean-field Hamiltonian, the partition function of a system is given by *Z* = T_r_[*e*^−*βH*^], where *β* = 1/*T* is the inverse temperature, T_r_ denotes the trace. Thus, the mean-field thermodynamic potential Ω = − ln *Z*/*β* in an FFLO state is given by,

(Equation 3) $$\begin{array}{lll} \Omega & = & \underset{k}{\sum }\left(\frac{\xi_{k}+\xi_{\text{Q}-\text{k}}}{2}-E_{k}\right)-\frac{\Delta^{2}}{U}\\ & - & T\underset{k}{\sum }\ln\left(1+e^{-\beta E_{k}^{\left(1\right)}}\right)\left(1+e^{-\beta E_{k}^{\left(2\right)}}\right). \end{array}$$

When *h* exceeds the critical value *h*_c_, the minimum of the thermodynamic potential shifts from *Q* = 0 to a finite *Q* > 0, signaling a phase transition from a conventional BCS superfluid to an FFLO superfluid. The parameters *μ*, Δ, and *Q* are consequently solved by using the stationary conditions, namely, *N* = −*∂*Ω/*∂μ*, *∂*Ω/*∂*Δ = 0, *∂*Ω/*∂Q* = 0, respectively. These three self-consistent equations can be obtained by

(Equation 4a) $$\begin{array}{ll} N & =\underset{k}{\sum }\left[1-D\left(k,T\right)\frac{\xi_{k}+\xi_{\text{Q}-\text{k}}}{2E_{k}}\right], \end{array}$$

(Equation 4b) $$\begin{array}{ll} -\frac{1}{U} & =\underset{k}{\sum }\frac{D\left(k,T\right)}{2E_{k}}, \end{array}$$

(Equation 4c) $$\begin{array}{ll} 0 & =\underset{k}{\sum }D\left(k,T\right)\left(1-\frac{\xi_{k}+\xi_{\text{Q}-\text{k}}}{2E_{k}}\right)\frac{\partial \xi_{\text{Q}-\text{k}}}{\partial Q}, \end{array}$$

where the function $D\left(k,T\right)=1-n_{F}\left(E_{k}^{\left(1\right)}\right)-n_{F}\left(E_{k}^{\left(2\right)}\right)$ with the Fermi distribution function *n*_*F*_(*x*) = 1/(*e*^*βx*^ + 1). To avoid numerical divergence caused by zeros of $E_{k}^{\left(1\right)}$, we set temperature to a typical low value *T* = 0.01*ϵ*_*F*_, approaching the zero-temperature limit. We use a *plane polar coordinate system* with *Q* aligned along the polar axis (**Q** = [*Q*, 0]) and *φ* as the polar angle. Moreover, *∂ξ*_**Q−k**_/*∂Q* = 2*t* sin(*Q* − *k* cos *φ*), where *φ* denotes the angle between **k** and **Q** and is integrated in the summation.

**Figure 1:**
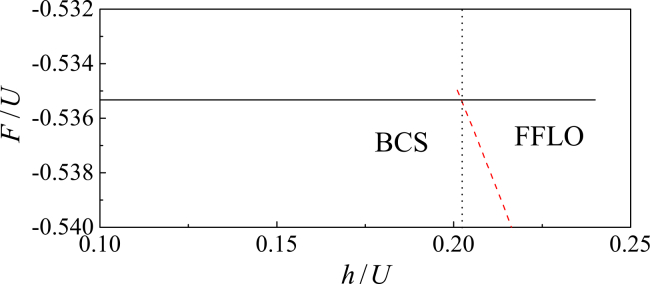
Mean-field free energy across the BCS-FFLO transition Free energy *F* = Ω + *μN* as a function of *h* for *t*/*U* = 0.3 and *n* = 0.8. The vertical dotted line marks the BCS-FFLO superfluid phase transition at *h*_c_/*U* = 0.202.

The ground state of the system is determined by minimizing the free energy, *F* = Ω + *μN*. In Figure 1, we plot *F* as a function of *h*.

Our calculations demonstrate that the ground state corresponds to a conventional BCS superfluid with *Q* = 0 at low *h*. When *h* > *h*_c_, the FFLO superfluid with finite *Q* has a lower free energy than the BCS superfluid, indicating a first-order phase transition. This make the FFLO superfluid become a more stable phase, in which pairing coexists with a finite magnetization. Both the chemical potential and the pairing gap change abruptly at *h*_c_. Near *h*_c_/*U* = 0.202, the pairing gap drops from Δ_BCS_/*U* = 0.29 in the BCS state to Δ_FFLO_/*U* = 0.097 in the FFLO side, where the corresponding COM momentum is *Q* = 0.82. Moreover, when the quantum fluctuations is considered, the pairing gap will be suppressed, which can be shown through QMC simulations.^61^ Owing to the bigger pairing gap in the mean-field level, the single-particle excitations have larger energy.

### Dynamical structure factor within the random phase approximation

The mean-field approximation neglects the quantum fluctuations. To incorporate the quantum fluctuations, the random phase approximation (RPA) provides a reliable framework for calculating response functions beyond the mean-field level.^93^^,^^94^^,^^95^ We briefly outline the main idea of RPA theory for investigating dynamical excitations. In an FFLO superfluid, four distinct density operators are relevant: the normal spin-up/down densities ${\hat{n}}_{1}=C_{↑}^{†}C_{↑}$ and ${\hat{n}}_{2}=C_{↓}^{†}C_{↓}$, together with the anomalous pairing operator and its complex conjugate ${\hat{n}}_{3}=C_{↓}C_{↑}$, ${\hat{n}}_{4}=C_{↑}^{†}C_{↓}^{†}$ that describe the Cooper pairing. These four densities are coupled by atomic interactions. A perturbation in any one of them induces fluctuations in the others. Within the linear response theory, a weak external perturbation potential *V*_ext_ perturbs the system and induces density fluctuations *δn*. The corresponding response function is defined as:

(Equation 5) $$\delta n=\chi V_{\text{ext}},$$

where $\delta n={\left[\delta n_{1},\;\delta n_{2},\;\delta n_{3},\;\delta n_{4}\right]}^{T}$ and $V_{\text{ext}}={\left[V_{1},\;V_{2},\;V_{3},\;V_{4}\right]}^{T}$. Within the RPA framework, an effective potential is defined as *V*_eff_ ≡ *V*_ext_ + *δV*^SC^. Here, *δV*^SC^ is the self-generated mean-field potential *δV*^SC^^93^ induced by the density fluctuations, given by

(Equation 6) $$\delta V^{\text{SC}}=U\int d^{2}r\left[\delta n_{4}{\hat{n}}_{3}+\delta n_{3}{\hat{n}}_{4}+\delta n_{1}{\hat{n}}_{2}+\delta n_{2}{\hat{n}}_{1}\right].$$

The density fluctuation *δn* is related to the effective potential *V*_eff_ by

(Equation 7) $$\delta n=\chi^{0}V_{\text{eff}},$$

where *χ*^0^ denotes the mean-field response function matrix, which can be readily computed. Beyond mean-field level, the response function *χ* within the RPA approach is connected to its mean-field counterpart *χ*^0^ by

(Equation 8) $$\chi \left(q,i\omega_{n}\right)=\frac{\chi^{0}\left(q,i\omega_{n}\right)}{\hat{1}-\chi^{0}\left(q,i\omega_{n}\right)UM_{I}}.$$

Here *M*_*I*_ = *σ*_0_ ⊗ *σ*_*x*_ is the direct product of unit matrix *σ*_0_ and the Pauli matrix *σ*_*x*_. The mean-field response function *χ*^0^ is easy to obtain, and its expression is a 4 × 4 matrix as,

(Equation 9) $$\chi^{0}\left(q,i\omega_{n}\right)=\left[\begin{array}{lllll} & \chi_{11}^{0} & \chi_{12}^{0} & \chi_{13}^{0} & \chi_{14}^{0}\\ & \chi_{21}^{0} & \chi_{22}^{0} & \chi_{23}^{0} & \chi_{24}^{0}\\ & \chi_{31}^{0} & \chi_{32}^{0} & \chi_{33}^{0} & \chi_{34}^{0}\\ & \chi_{41}^{0} & \chi_{42}^{0} & \chi_{43}^{0} & \chi_{44}^{0} \end{array}\right].$$

The dimension of *χ*^0^ reflects the coupling among the four density channels. These 16 matrix elements are given by the density-density correlation functions derived from the defined Green’s functions defined earlier. For example, $\chi_{12}^{0}=-\left\langle T_{\tau }\psi_{↑}^{†}\left(r,\tau \right)\psi_{↑}\left(r,\tau \right)\psi_{↓}^{†}\left(r^{\prime },\tau^{\prime }\right)\psi_{↓}\left(r^{\prime },\tau^{\prime }\right)\right\rangle$, which, after contraction via Wick’s theorem, reduces to $\chi_{12}^{0}=-\Gamma^{†}\left(r-r^{\prime },\tau -\tau^{\prime }\right)\Gamma \left(r^{\prime }-r,\tau^{\prime }-\tau \right)$. Owing to the symmetries of the system, only nine matrix elements are independent, i.e., $\chi_{12}^{0}=\chi_{21}^{0}=-\chi_{33}^{0}=-\chi_{44}^{0}$, $\chi_{31}^{0}=\chi_{14}^{0}$, $\chi_{32}^{0}=\chi_{24}^{0}\;\chi_{42}^{0}=\chi_{23}^{0}$, $\chi_{41}^{0}=\chi_{13}^{0}$. Their explicit analytical forms are provided in the supplemental information.

The total density (spin) response function *χ*_*D*_(*χ*_*S*_) is defined as *χ*_*D*_ ≡ *χ*_11_ + *χ*_12_ + *χ*_21_ + *χ*_22_(*χ*_*S*_ ≡ *χ*_11_ − *χ*_12_ − *χ*_21_ + *χ*_22_). According to the fluctuation-dissipation theory, the density (spin) dynamical structure factor *S*_*D*_(**q**, *ω*)(*S*_*S*_(**q**, *ω*)) is given by,

(Equation 10) $$S_{D/S}\left(q,\omega \right)=-\frac{1}{\pi }\frac{1}{1-e^{-\omega /T}}Im\chi_{D/S}\left(q,i\omega_{n}\to \omega +i\delta \right),$$

where **q** and *ω* denote the transferred momentum and energy, respectively. The parameter *δ* is a small positive number introduced in numerical calculations, typically set to *δ* = 0.003.

### Results at half-filling

**Figure 2:**
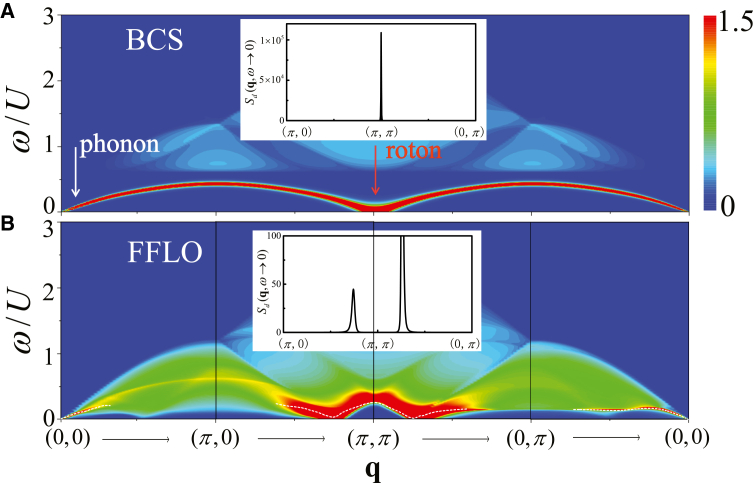
Density response across the BCS-FFLO transition Density dynamical structure factor *S*_*D*_(**q**, *ω*) along [0, 0] → [*π*, 0] → [*π*, *π*] → [0, *π*] → [0, 0] in the Brillouin zone with *n* = 1.0. (A) BCS superfluid at *h*/*U* = 0.19. (B) FFLO superfluid at *h*/*U* = 0.1978. The white dashed lines exhibit the solution of ReΣ = 0 as a function of **q**.

**Figure 3:**
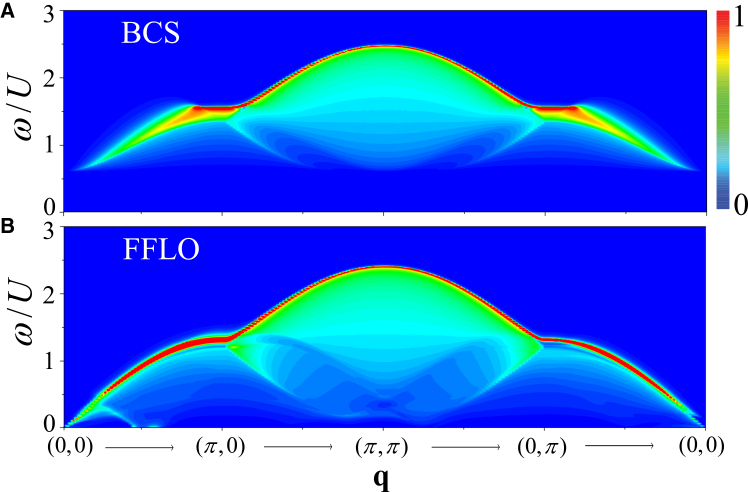
Spin response across the BCS-FFLO transition Spin dynamical structure factor *S*_*S*_(**q**, *ω*) along the same momentum path and under the same density condition as in Figure 2. (A) BCS superfluid at *h*/*U* = 0.19. (B) FFLO superfluid at *h*/*U* = 0.1978.

By continuously increasing the Zeeman field strength across the critical value *h*_*c*_, the system undergoes a phase transition from a BCS to an FFLO superfluid, resulting in notable changes in both the collective modes and single-particle excitations. In Figure 2, we plot the density dynamical structure factor *S*_*D*_(**q**, *ω*) along the high-symmetry path of the first Brillouin zone (BZ), [0, 0] → [*π*, 0] → [*π*, *π*] → [0, *π*] → [0, 0], for (1) *h*/*U* = 0.19 (BCS superfluid) and (b) *h*_*c*_/*U* = 0.1978 (FFLO superfluid), respectively. The corresponding spin dynamical structure factor *S*_*S*_(**q**, *ω*) is shown in Figure 3 under the same parameters. Here, we fix the density at *n* = 1 and the hopping strength at *t*/*U* = 0.3.

Obviously, both *S*_*D*_(**q**, *ω*) and *S*_*S*_(**q**, *ω*) change dramatically across the transition from a BCS to an FFLO superfluid, which is mainly attributed to the discontinuous decrease of the pairing gap and the emergence of a finite COM momentum **Q** at the transition point. In the BCS superfluid, *S*_*D*_(**q**, *ω*) exhibit a sharp collective phonon mode starting from **q** = [0, 0], and a roton mode starting from **q** = [*π*, *π*] exhibit a “V” form. When entering the FFLO superfluid, the collective phonon mode mixes with the gapless single-particle excitations and the roton mode has a “W” structure. Moreover, at the high energy region, *S*_*S*_(**q**, *ω*) exhibits a conventional magnetic mode in both the BCS and the FFLO superfluids.

For the BCS state, the single-particle excitations are gapped and the continuum starts at *ω* ≥ 2Δ. However, for the FFLO superfluid, the quasiparticle energy bands pass through the Fermi energy, giving rise to a Bogoliubov Fermi surface. Another low-energy collective mode associated with the Bogoliubov quasiparticle (bogolon) emerges around **q** = [0, 0] in the spin channel. Consequently, the gapless single-particle excitations compete with these collective modes and damp them. Moreover, for an FFLO superfluid, the quasiparticle energy band structure exhibits asymmetry relative to the **Q** direction,^17^^,^^27^^,^^100^^,^^101^^,^^102^ leading to the anisotropic dynamical excitations compared with the BCS case.

#### Phonon and bogolons

At small transferred momenta near Γ(**q** = [0, 0]), the dynamical structure factors exhibit a sharp narrow peak with a linear dispersion emerging from zero energy, indicating appearance of the collective modes. The phonon mode originates from the spontaneously U(1) phase symmetry breaking of order parameter in the superfluid state.^103^^,^^104^ In the BCS superfluid, the phonon mode is always well separated from the single-particle continuum. In the FFLO superfluid, however, the phonon mode merges into the single-particle excitation continuum and undergoes spectral broadening through the scattering with the single-particle excitations. In addition to the Cooper pairs, the existence of unpaired, strong spin-polarized atoms near the Bogoliubov Fermi surface give rise to the collective bogolon mode, enhancing the competition between the single-particle excitations and the collective modes.

**Figure 4:**
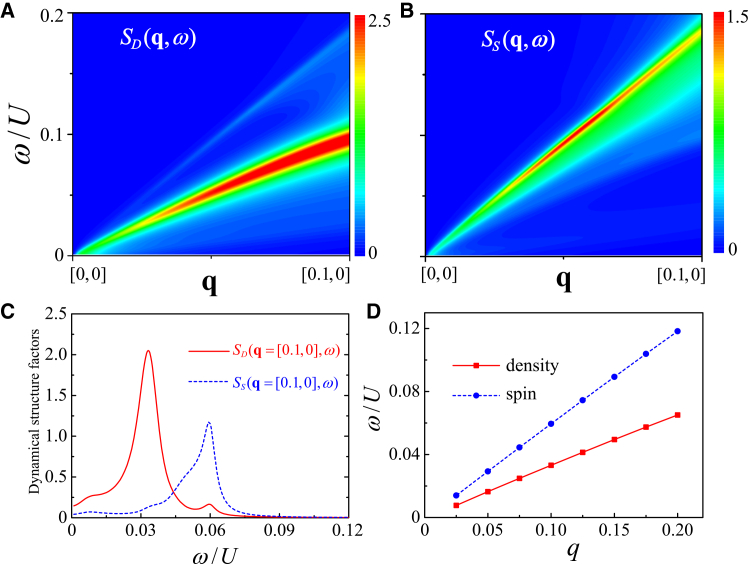
Low-momentum phonon and bogolon modes in the FFLO superfluid Dynamical structure factors and extracted collective-mode dispersions in the low transferred momentum region. The parameters are *h*/*U* = 0.1978, *t*/*U* = 0.3, and *n* = 1. (A) Density dynamical structure factor *S*_*D*_(**q**, *ω*) along [0, 0] → [*π*, 0]. (B) Spin dynamical structure factor *S*_*S*_(**q**, *ω*) along [0, 0] → [*π*, 0]. (C) *S*_*D*_(**q** = [0.1, 0], *ω*) (red solid line) and *S*_*S*_(**q** = [0.1, 0], *ω*) (blue dashed line) as functions of *ω*. (D) Peak positions of the two collective modes extracted from *S*_*D*_(**q**, *ω*) and *S*_*S*_(**q**, *ω*).

To clearly elucidate these two collective modes in the FFLO superfluid, the zoomed-in *S*_*D*_(**q**, *ω*) and *S*_*S*_(**q**, *ω*) in the low-momentum region along [0, 0] → [0.1, 0] are shown in Figures 4A and 4B, respectively. Figure 4C plot *S*_*D*_(**q** = [0.1, 0], *ω*) (red solid line) and *S*_*S*_(**q** = [0.1, 0], *ω*) (blue dashed line) as a function of *ω*. The extracted peak positions of them are summarized in Figure 4D.

Obviously, Figures 4A and 4B exhibit two distinct linear collective modes, respectively. A sharp peak in *S*_*D*_(**q** = [0.1, 0], *ω*) at *ω*/*U* = 0.033 corresponds to the collective phonon mode. Another peak found in *S*_*S*_(**q** = [0.1, 0], *ω*) at *ω*/*U* = 0.06 is a collective bogolon mode. The slope of a collective mode defines the corresponding speed: the sound speed *c*_s_ in the density channel and the bogolon speed *c*_b_ in the spin channel. The unpaired atoms near the Fermi surface are strongly polarized, which explains why the bogolon mode exhibits a stronger signal in the spin dynamical excitations. The magnitude of these two speeds can be extracted by fitting the position of the *δ*-like peak of dynamical structure factors at a small transferred momentum, *c*_s∕b_ = *ω*/*q*. The bogolon speed *c*_b_/*U* = 0.60 significantly exceeds that sound speed *c*_s_/*U* = 0.33. These collective modes can be analyzed through the RPA formula. From Equation 8, the collective modes correspond to the poles of the response function *χ*, i.e., zeros of the denominator det$\left[\hat{1}-\chi^{0}\left(q,i\omega_{n}\right)UM_{I}\right]$, which is simplified as $\Sigma =\left[\chi_{34}^{0}\chi_{43}^{0}-{\left(\chi_{12}^{0}\right)}^{2}\right]$. Following our earlier method,^52^ the dispersion of main collective modes is determined by solving the self-consistent equation, ReΣ = 0. The solution of ReΣ = 0, marked by the white dashed lines, are shown in Figure 3B.

#### Roton mode and COM momentum measurement protocol

For the BCS superfluid, the roton mode emerges at the momentum point **q** = [*π*, *π*]. Its emergence can be attributed to the breaking of a global pseudospin *SU*(2) symmetry.^105^^,^^106^ Owing to the particle-hole symmetry at half-filling, the fluctuations of superfluid and CDW become degenerate, leading to the appearance of a gapless roton mode. In contrast, for the FFLO superfluid, the minimum of the roton mode is not localized at a single momentum but forms a ring in the BZ centered at **q** = [*π*, *π*], with a radius *Q* = |**Q**|. The roton mode is closely related to the nesting of the Fermi surface. In the BCS state with *Q* = 0, the nesting vector **q**_**nest**_ = [*π*, *π*] at half-filling determines the position of the roton mode. In the FFLO state, however, the Fermi surface becomes more complex due to the presence of the Zeeman field. The particle numbers for different spins become unequal, leading to a separation between the spin-up and spin-down Fermi surfaces. The separation distance is given by *Q*.

**Figure 5:**
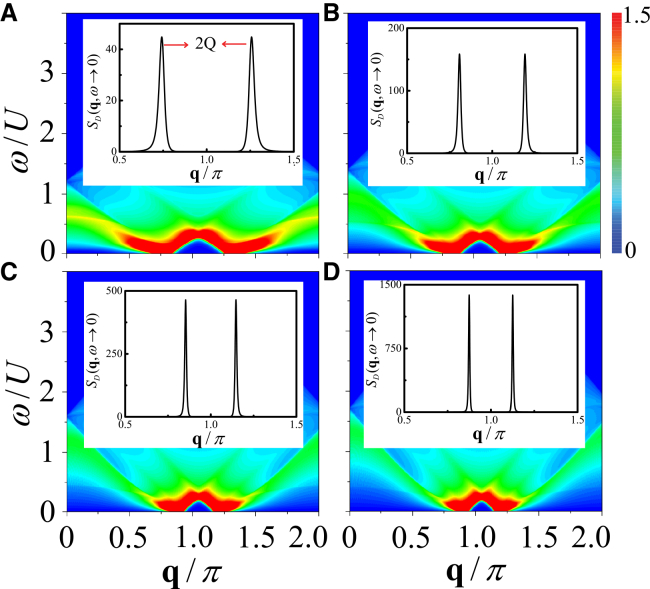
Roton-mode evolution with hopping strength Density dynamical structure factor *S*_*D*_(**q**, *ω*) along [*π*, 0] → [*π*, *π*] → [*π*, 2*π*] for different hopping strengths. (A) *t*/*U* = 0.3. (B) *t*/*U* = 0.35. (C) *t*/*U* = 0.4. (D) *t*/*U* = 0.43.

The ring structure of the roton mode depends sensitively on the interaction strength. In Figure 5, we present the contour plots of *S*_*D*_(**q**, *ω*) along the path [*π*, 0] → [*π*, *π*] → [*π*, 2*π*] in the BZ for various hopping strengths.

**Figure 6:**
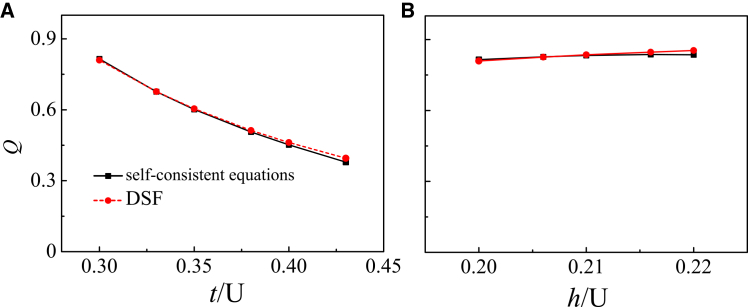
Roton-based extraction of the Cooper-pair momentum Center-of-mass momentum *Q* obtained from the self-consistent calculations (black line) and from the dynamical structure factor (red line). (A) *Q* as a function of hopping strength *t*. (B) *Q* as a function of Zeeman field strength *h*.

As *t*/*U* increases, the distance by which the roton mode deviates from [*π*, *π*] decreases and becomes approximately equal to the magnitude of COM momentum *Q*. To clearly demonstrate the connection between the roton mode and *Q*, we extract the displacement of the roton mode from Figure 5, and plot *Q* (red line) as a function of hopping strength *t* in Figure 6, comparing it with the *Q* obtained from the self-consistent calculations (black line).

Our results show that the distance of the roton mode deviating from [*π*, *π*] exhibits an almost identical trend for different values of *t* and *h*. These results provide an important insight: experimentally, one can directly measure the magnitude of *Q* by detecting the displacement of the roton mode from [*π*, *π*] in a half-filled lattice system.

#### Angular dependence of dynamical excitations

**Figure 7:**
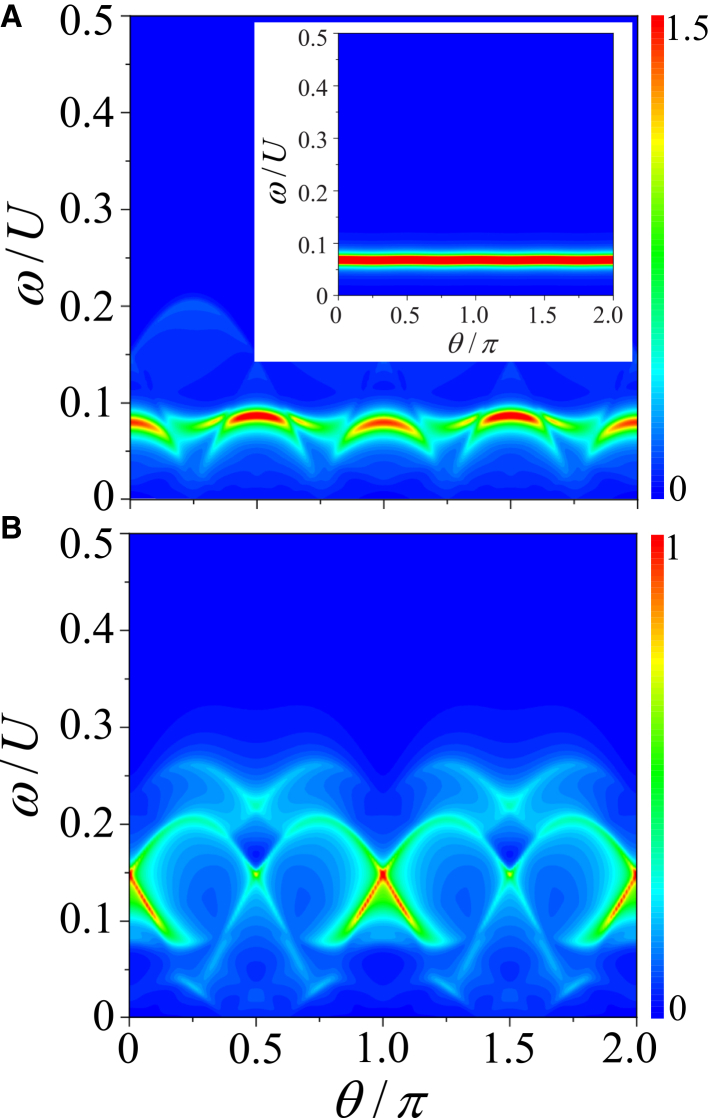
Angular anisotropy of low-momentum dynamical excitations Angular dependence of dynamical structure factors in an FFLO superfluid as a function of *θ* from 0 to 2*π*, with *q* = 0.08*π*, *n* = 1.0, and *h*/*U* = 0.1978. (A) Density dynamical structure factor *S*_*D*_(*q* = 0.08*π*, *ω*). The inset shows the corresponding *S*_*D*_(*q* = 0.08*π*, *ω*) in the BCS superfluid for *h*/*U* = 0.19. (B) Spin dynamical structure factor *S*_*S*_(*q* = 0.08*π*, *ω*).

To show the anisotropic behavior more clearly, we calculate the angular dependence of the dynamical structure factors at a small transferred momentum. The contour plots of *S*_*D*_(*q* = 0.08*π*, *ω*) and *S*_*S*_(*q* = 0.08*π*, *ω*) as a function of *θ* are shown in Figure 7, spanning the full angular range.

In an FFLO superfluid, both *S*_*D*_(*q*, *ω*) and *S*_*S*_(*q*, *ω*) as a function of *θ* exhibit an anisotropic behavior, which can be attributed to the finite **Q**. Obviously, the strength of the phonon mode evolves with a period of *π* in angle, contrasting sharply with the behavior in a BCS superfluid, where the phonon mode remains nearly constant. Furthermore, the slope of the phonon mode related to the sound speed *c*_s_ also varies with angle. Notably, unlike a BCS superfluid where the single-particle excitations are gapped by the superfluid gap, the gapless single-particle excitations competes intensely with the phonon mode, and even destroy it at certain angles. Similarly, *S*_*S*_(**q**, *ω*) displays angular anisotropy, and the bogolon mode is also destroyed by the single-particle excitations at certain angles.

#### Single-particle excitations

The energy spectra are reconstructed under the Zeeman field, leading to the complex single-particle excitations in an FFLO superfluid, particularly in manifesting the Cooper pair-breaking mechanism. Based on the two quasiparticle spectra $E_{k}^{\left(1\right)}$, $E_{k}^{\left(2\right)}$, the dynamical excitations take the following four forms: $\hbar \omega_{kq}=E_{\mathrm{k+q}}^{\left(1\right)}-E_{k}^{\left(1\right)},E_{\mathrm{k+q}}^{\left(2\right)}-E_{k}^{\left(2\right)},E_{\mathrm{k+q}}^{\left(1\right)}+E_{k}^{\left(2\right)},E_{\mathrm{k+q}}^{\left(2\right)}+E_{k}^{\left(1\right)}$, respectively. These four kinds of excitations are expressed by the kernel functions *L*_1_(**k**, **q**, *iω*_*n*_), *L*_2_(**k**, **q**, *iω*_*n*_), *L*_3_(**k**, **q**, *iω*_*n*_), and *L*_4_(**k**, **q**, *iω*_*n*_) in the supplemental information, where *L*_1_ and *L*_4_ are the intra-band excitations while *L*_2_ and *L*_3_ are the inter-band excitations. Notably, the minima of intra-band channel remain gapless owing to the Bogoliubov Fermi surface while the inter-band channel is gapped.

**Figure 8:**
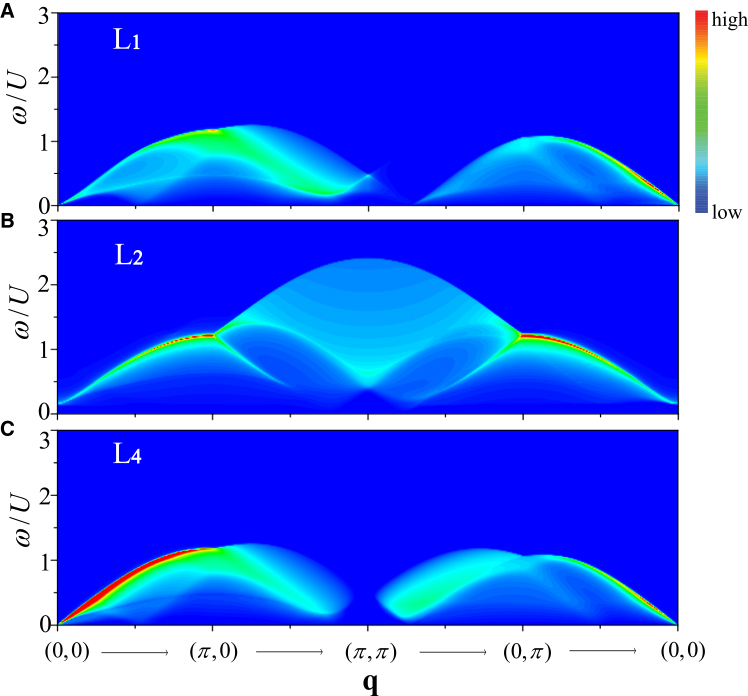
Single-particle excitation channels in the density response Partial color maps of the density dynamical structure factor *S*_*D*_(*q*, *ω*) along [0, 0] → [*π*, 0] → [*π*, *π*] → [0, *π*] → [0, 0] for *h*/*U* = 0.1978 and *n* = 1.0. (A) Contribution from the kernel function *L*_1_(**k**, **q**, *iω*_*n*_). (B) Contribution from the kernel function *L*_2_(**k**, **q**, *iω*_*n*_). (C) Contribution from the kernel function *L*_4_(**k**, **q**, *iω*_*n*_).

To identify the role of each channel, the *S*_*D*_(*q*, *ω*) at *h* = 0.2 is calculated in Figure 8 when only one of kernel functions is considered at a time for (1) *L*_1_(**k**, **q**, *iω*_*n*_), (2) *L*_2_(**k**, **q**, *iω*_*n*_), and (3) *L*_4_(**k**, **q**, *iω*_*n*_).

It is shown that the low-energy gapless single-particle excitations mainly originate from the intra-band excitations (*L*_1_ and *L*_4_) while the inter-band contribution *L*_3_ remains gapped and determines the high-energy single-particle excitations, especially for the vicinity around **q** = [*π*, *π*]. Moreover, based on our calculations, the contribution from *L*_3_(**k**, **q**, *iω*_*n*_) term is very weak and is therefore omitted here.

**Figure 9:**
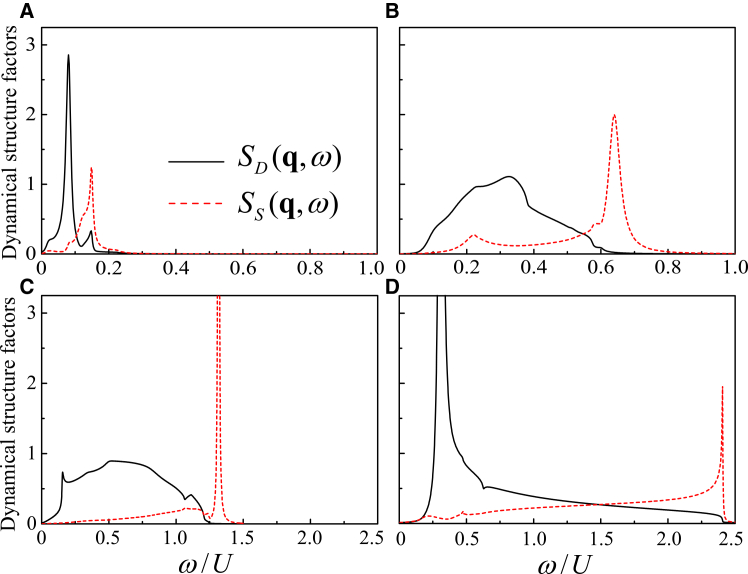
Density and spin spectra at representative momenta Line cuts of *S*_*D*_(**q**, *ω*) (black solid line) and *S*_*S*_(**q**, *ω*) (red dashed line) as functions of *ω* for *h*/*U* = 0.1978 and *t*/*U* = 0.3. (A) **q** = [0.08*π*, 0]. (B) **q** = [0.32*π*, 0]. (C) **q** = [0, *π*]. (D) **q** = [*π*, *π*].

Based on the separate calculation results above, we further calculate the energy dependence of *S*_*D*_(**q**, *ω*) and *S*_*S*_(**q**, *ω*) at several typical transferred momenta in the BZ. The results for *S*_*D*_(**q**, *ω*) (black solid line) and *S*_*S*_(**q**, *ω*) (red dashed line) are plotted in Figure 9.

At small *q*, a strong, sharp phonon peak appears in the low-energy region, while three kinds of the single-particle excitations at the larger energies are comparatively weak. Owing to the presence of the gapless single-particle excitations, the phonon (bogolon) peak is suppressed and exhibits a finite width, as the phonon (bogolon) mode is pushed into the single-particle excitation region and competes with them. In image (c), the conventional magnetic mode is separated from the dominant single-particle excitation continuum, producing a sharp magnetic collective peak in *S*_*S*_(**q**, *ω*). In image (d), a low-energy sharp peak emerges at *q* = [*π*, *π*] around *ω*/*U* = 0.31, reflecting the combined effect of the single-particle excitation and roton-related collective excitations. A higher-energy peak near *ω*/*U* = 2.41 signals the conventional magnetic mode. Moreover, this excitation gap is doping dependent and will be discussed in the following section.

### Doping dependence of dynamical excitations

**Figure 10:**
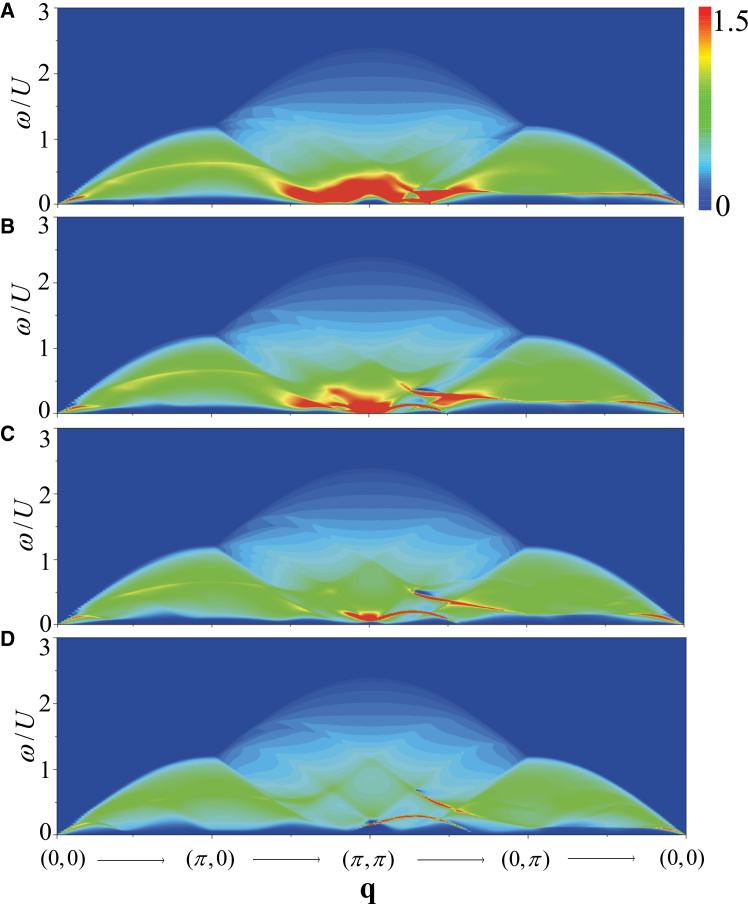
Doping dependence of density dynamical excitations Color maps of the density dynamical structure factor *S*_*D*_(**q**, *ω*) with *t*/*U* = 0.3 for different densities. (A) *n* = 0.9. (B) *n* = 0.8. (C) *n* = 0.7. (D) *n* = 0.6.

**Figure 11:**
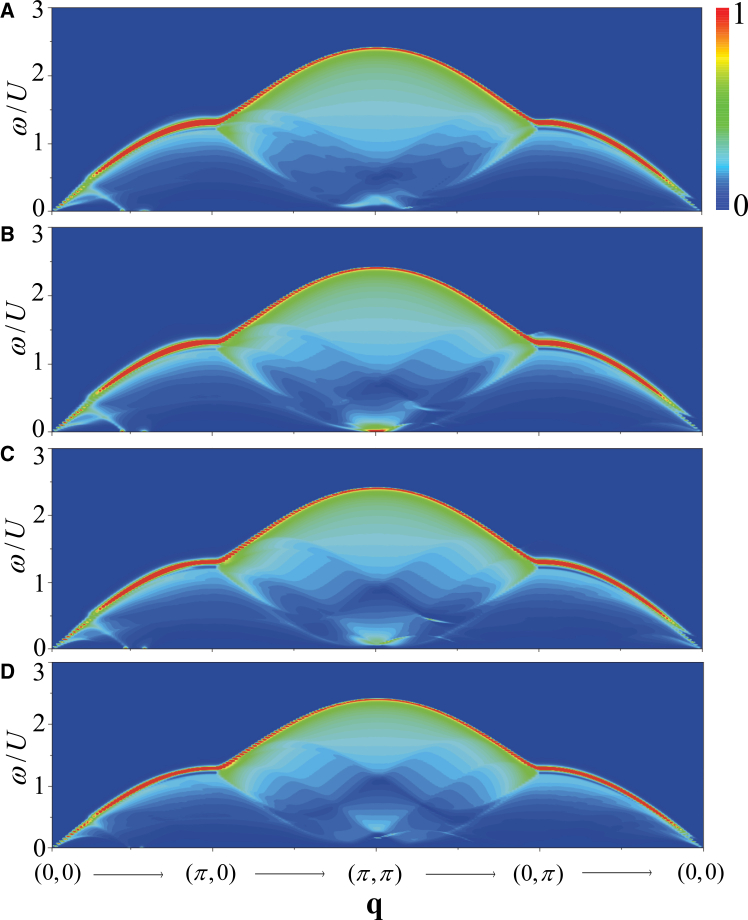
Doping dependence of spin dynamical excitations Color maps of the spin dynamical structure factor *S*_*S*_(**q**, *ω*) with *t*/*U* = 0.3 for different densities. (A) *n* = 0.9. (B) *n* = 0.8. (C) *n* = 0.7. (D) *n* = 0.6.

The doping can alter the chemical potential, the pairing gap and the quasiparticle spectra, leading to the modification of the dynamical excitations. Therefore, we discuss the doping dependent of the dynamical excitations in the FFLO superfluid. In Figure 10, we plot *S*_*D*_(**q**, *ω*) along the high-symmetry directions of the BZ for (1) *n* = 0.9, (2) *n* = 0.8, (3) *n* = 0.7, and (4) *n* = 0.6 at the BCS-FFLO transition point *h*_*c*_ with *t*/*U* = 0.3. In Figure 11, *S*_*S*_(**q**, *ω*) as a function of doping are plotted with the same parameters.

**Figure 12:**
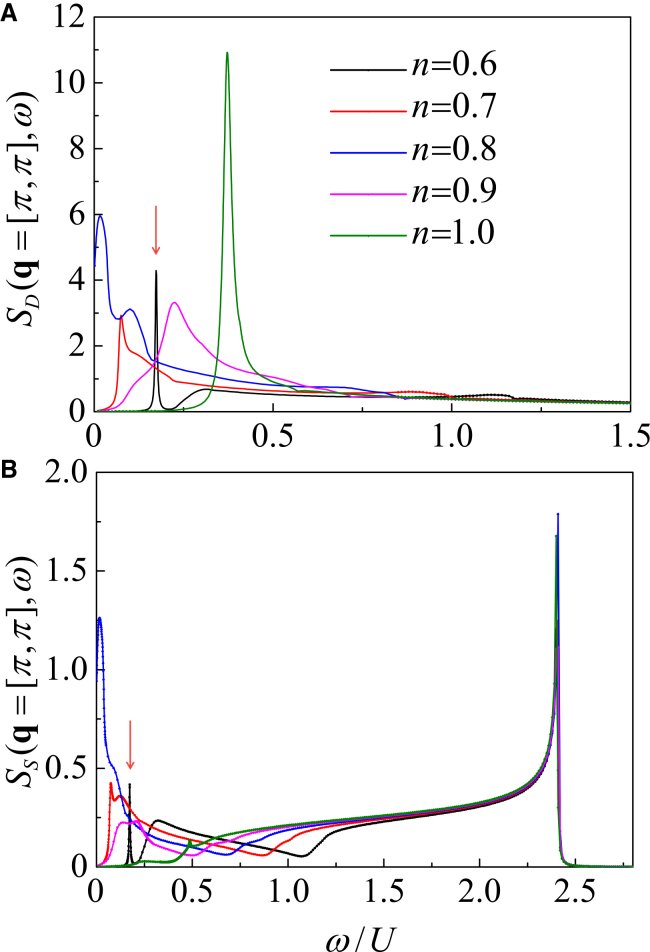
Doping evolution at the antiferromagnetic momentum Dynamical structure factors at **q** = [*π*, *π*] as functions of the density *n* at the BCS-FFLO transition point *h*_c_. (A) Density dynamical structure factor *S*_*D*_(**q** = [*π*, *π*], *ω*). (B) Spin dynamical structure factor *S*_*S*_(**q** = [*π*, *π*], *ω*).

To study the dynamical excitations at **q** = [*π*, *π*], we study the doping dependence of dynamical structure factors. As shown in Figure 12, the dynamical structure factors in the FFLO superfluid are investigated for (1) *S*_*D*_(**q**, *ω*) and (2) *S*_*S*_(**q**, *ω*).

Both *S*_*D*_(**q** = [*π*, *π*], *ω*) and *S*_*S*_(**q** = [*π*, *π*], *ω*) are strongly doping dependent. As *n* decreases (the doping concentration increases), the excitation gap initially narrows, vanishing around *n* = 0.8, and then reopens at lower *n*, exhibiting an open-close-reopen behavior. In particular, at *n* = 0.6, the collective mode and the single-particle excitations band are well separated, indicating that the dynamical excitations at **q** = [*π*, *π*] consists of a sharp collective mode (marked by the arrow) and a broad continuum of the single-particle excitations. Furthermore, in Figure 12B, the sharp peaks observed around *ω*/*U* = 2.406 correspond to the conventional magnetic mode, whose positions remain independent of doping concentration.

## Discussion

Following the phase transition from a conventional BCS superfluid to an FFLO superfluid, the dynamical excitations exhibits several novel features, such as the emergence of two distinct collective modes around **q** = [0, 0] and a circular roton mode centered at **q** = [*π*, *π*]. In addition to the phonon, when the system enters the FFLO superfluid, the energy bands cross the Fermi energy, giving rise to both the gapless single-particle excitations and bogolon mode. This leads to the intense competitions between the single-particle excitations and the collective modes, which shorten or even eliminate the lifetime of the collective modes, making them challenging to observe experimentally. In a conventional BCS superfluid, the Meissner effect ensures an equal population of the spin-up and spin-down atoms, resulting in a non-polarized state. In contrast, in an FFLO state, the applied Zeeman field induces a population imbalance between spin-up and spin-down atoms on the Fermi surface, leading to a net spin polarization and the appearance of the bogolon mode.

In an FFLO superfluid, the roton mode forms a ring structure centered at **q** = [*π*, *π*] with a radius equal to COM momentum *Q*, indicating *Q* directly modifies the dynamical excitations. The finite *Q* also indicates that the FFLO superfluid exhibits a different symmetry-breaking physics compared to the BCS superfluid. In our previous work on a BCS superfluid at half-filling on an optical lattice (where *Q* = 0), the roton mode is found to be gapless and located precisely at *q* = [*π*, *π*] owing to the particle-hole symmetry. In the FFLO superfluid studied here, however, the particle-symmetry is broken, causing the movement of the roton mode from the [*π*, *π*].

In this paper, for the pairing gap, we adopt the plane wave form (FF-type), Δ(**r**) = Δ*e*^*i***Q**⋅**r**^, which breaks the time-reversal symmetry while preserving the translational symmetry. In fact, there is another form of the pairing gap, namely, the double-**Q** standing wave form (LO-type) Δ(**r**) = Δ_0_ cos[**Q** ⋅ **r**],^107^^,^^108^^,^^109^ which breaks the translational symmetry but preserves the time-reversal symmetry. Due to the breaking of the translational symmetry, a new gapless Goldstone mode emerges, intensifying the competition between the single-particle excitations and the collective modes.^91^^,^^110^ This new gapless Goldstone mode may significantly affect the roton mode with the ring-shaped feature discussed in this paper. However, the research method for the LO state differs from that for the FF state. For the LO state, exhibiting the breaking of the translational symmetry, the dynamical excitations need to be studied in a finite-size system, in contrast to the infinite system considered in this paper. In future work, we will develop a method to study the dynamical structure factor of LO-type superfluids within a finite-size system.

### Limitations of the study

Although this work provides a detailed characterization of dynamical excitations and proposes a roton-based protocol for measuring the center-of-mass (COM) momentum in a two-dimensional FFLO superfluid, several limitations should be acknowledged.

First, our theoretical framework relies on the random phase approximation (RPA) to treat the interaction effects and collective modes. While RPA is a standard and effective tool for describing collective excitations in the weak-to-intermediate coupling regime, its quantitative accuracy may diminish in strongly correlated systems where the attractive interaction |*U*| is large. In such strong-coupling regimes, higher-order vertex corrections and self-energy effects beyond the RPA level could significantly modify the excitation energy scales and spectral weights.

Second, the proposed roton-based protocol for determining the COM momentum *Q* is currently validated primarily for the half-filled case. At half-filling, the system possesses specific symmetries that allow for a clear identification of the roton minimum’s displacement from the (*π*, *π*) point. For systems away from half-filling, the deformation of the Fermi surface and the increased complexity of the quasiparticle continuum may pose challenges to the uniqueness and visibility of the roton feature, necessitating further investigation to generalize the method to arbitrary filling factors.

Furthermore, the current analysis is conducted at zero temperature. In experimental settings with ultracold atoms, finite-temperature thermal fluctuations can lead to the damping of collective modes and potentially influence the phase stability of the FFLO state. Lastly, this study is restricted to a square lattice geometry. The exploration of dynamical signatures and pairing characteristics in other lattice types, such as triangular or hexagonal lattices, remains an important direction for future theoretical and experimental work.

## Resource availability

### Lead contact

Further information and requests for resources should be directed to and will be fulfilled by the lead contact, Huaisong Zhao (hszhao@qdu.edu.cn).

### Materials availability

This study did not generate new biological materials or unique physical samples.

### Data and code availability

• The numerical source data underlying the figures and the processed data supporting the findings of this study have been deposited in the Mendeley Data repository and are publicly available at: https://data.mendeley.com/datasets/t7sjrjfvhf/1.
• The original Fortran code used for the mean-field and random-phase-approximation calculations has been deposited in the Mendeley Data repository and is publicly available at: https://data.mendeley.com/datasets/t7sjrjfvhf/1.
• Any additional information required to reanalyze the data reported in this paper is also included in the Mendeley Data repository at: https://data.mendeley.com/datasets/t7sjrjfvhf/1.

## Acknowledgments

The authors would like to thank Feng Yuan for helpful discussions. This work was supported by the funds from the Research Foundation of Yanshan University under grant no. 8190448 (S.T.), the 10.13039/501100001809National Natural Science Foundation of China under grant nos. U23A2073 (P.Z.) and 11547034 (H.Z.).

## Author contributions

H.Z. conceived the project. The model, RPA theory, and physical explanation were finished by H.Z., T.M., and P.Z. The fortran codes and most figures were finished by S.T. and H.Z. The sound speed was calculated by J.S. H.Z. and P.Z. wrote the manuscript with inputs from all coauthors.

## Declaration of interests

The authors declare no competing interests.

## STAR★Methods

### Key resources table

**Table:** 

REAGENT or RESOURCE	SOURCE	IDENTIFIER
**Deposited data**		

Numerical source data underlying the main figures	This paper	https://data.mendeley.com/datasets/t7sjrjfvhf/1

**Software and algorithms**		

Fortran2016	Intel	https://www.intel.com/content/www/us/en/developer/tools/oneapi/fortran-compiler.html
Origin2018	OriginLab	https://www.originlab.com/

### Method details

#### Model Hamiltonian

We consider a spin-polarized two-dimensional attractive Fermi-Hubbard model on a square optical lattice under an effective Zeeman field. In momentum space, the Hamiltonian is written as

$$H=\Sigma_{k,\sigma }\xi_{k\sigma }c_{k\sigma }^{†}c_{k\sigma }-U\Sigma_{k}c_{k↑}^{†}c_{Q-k↓}^{†}c_{Q-k↓}c_{k↑,}$$

where $\xi_{k\sigma }=\varepsilon_{k}-h\sigma_{z}$, $\varepsilon_{k}=-Zt\gamma_{k}-\mu$, $\gamma_{k}=\left(\cos\;k_{x}+\cos\;k_{y}\right)/2$, and $\sigma_{z}=+1\left(-1\right)$ for spin ↑ (↓). Here μ is the average chemical potential, h is the effective Zeeman field, U > 0 is the on-site attractive interaction, and Q is the center-of-mass momentum of Cooper pairs. For the square lattice, the coordination number is Z = 4. Throughout the calculation, U is taken as the unit of energy and the lattice constant a0 as the unit of length.

#### Mean-field Green’s functions

For the Fulde-Ferrell state, the pairing order parameter is assumed to have the plane-wave form Δ(r)=Δexp(iQ·r). Within mean-field theory, the interaction term is decoupled in the pairing channel, yielding the mean-field Hamiltonian

$$H_{MF}=\Sigma_{k,\sigma }\xi_{k\sigma }c{†}_{k\sigma }c_{k\sigma }{+\Delta }^{2}/U-\Sigma_{k}\left(\Delta c_{k↑}^{†}c_{Q-k↓}^{†}+\Delta *c_{Q-k↓}c_{k↑}\right)\text{.}$$

We introduce the spin-resolved normal Green’s functions G↑(k,τ−τ′), G↓(k,τ−τ′), and the anomalous singlet pairing Green’s function $\Gamma^{†}\left(k,\tau -\tau^{\prime }\right)$. Using the equations-of-motion method, we obtain the mean-field propagators for the spin-up, spin-down, and anomalous channels. The coherence factors are $U_{k}^{2}=1/2\left[1+\left(\xi_{k}+\xi_{Q-k}\right)/\left(2E_{k}\right)\right]$ and $V_{k}^{2}=1/2\left[1-\left(\xi_{k}+\xi_{Q-k}\right)/\left(2E_{k}\right)\right]$, with $E_{k}=\sqrt{\left[\left(\xi_{k}+\xi_{Q-k}\right)2/4+\Delta^{2}\right]}$. The quasiparticle branches are $E_{k}^{\left(1\right)}=E_{k}+\left(\xi_{k}-\xi_{Q-k}\right)/2-h$ and $E_{k}^{\left(2\right)}=E_{k}-\left(\xi_{k}-\xi_{Q-k}\right)/2+h$. In the FFLO state, $E_{k}^{\left(1\right)}$ can cross the Fermi energy and generate a Bogoliubov Fermi surface, which is essential for the low-energy bogolon excitations discussed in the main text.

#### Self-consistent FFLO solution

The mean-field thermodynamic potential is evaluated from the partition function and written as

Ω=Σk[ξk+ξQ−k2−Ek]−Δ2U−TΣkln[(1+e(−βEk(1)))(1+e(−βEk(2)))].

The chemical potential μ, pairing gap Δ, and Cooper-pair momentum Q are determined self-consistently from N=−∂Ω/∂μ, ∂Ω/∂Δ=0, and ∂Ω/∂Q=0. The corresponding number, gap, and momentum equations are solved iteratively. To avoid numerical divergence associated with zeros of $E_{k}^{\left(1\right)}$, calculations are carried out at a low but finite temperature $T=0.01\varepsilon_{F}$, which approximates the zero-temperature limit. In the numerical implementation, Q is taken along the x direction, Q=[Q,0], and the polar angle between k and Q is included in the momentum summation. The ground state is identified by minimizing the free energy F=Ω+μN and comparing the conventional BCS solution (Q=0) with the FFLO solution (Q>0).

#### Response functions in RPA

Quantum fluctuations beyond mean field are incorporated within the random phase approximation (RPA). Four coupled density operators are retained: the spin-up density $n_{1}=c_{↑}^{†}c_{↑}$, the spin-down density $n_{2}=c_{↓}^{†}c_{↓}$, the anomalous pairing field $n_{3}=c_{↓}c_{↑}$, and its conjugate $n_{4}=c_{↓}^{†}c_{↑}^{†}$. Under a weak external perturbation $V_{ext}$, the induced density fluctuation satisfies $\delta n=\chi V_{ext}$. The self-generated mean-field potential induced by density fluctuations is included through a self-consistent potential $\delta V^{SC}$, leading to the RPA response matrix

$$\chi \left(q,i\omega_{n}\right)=\chi^{0}\left(q,i\omega_{n}\right){\left[I-\chi^{0}\left(q,i\omega_{n}\right)UM_{I}\right]}^{-1}\text{,}$$

where $M_{I}=\sigma_{0}⊗\sigma_{x}$ and $\chi^{0}$ is the mean-field response matrix. Because of symmetry, nine matrix elements of χ0 are independent. These matrix elements are computed from the normal and anomalous Green’s functions via Wick contraction. The total density and spin response functions are defined as ${\chi \;}_{D}={\;\chi }_{11}+{\;\chi }_{12}+{\;\chi }_{21}+{\;\chi }_{22}$ and ${\chi \;}_{S}={\;\chi }_{11}-{\;\chi }_{12}-{\;\chi }_{21}+{\;\chi }_{22}$, respectively.

#### Dynamical structure factors

The density and spin dynamical structure factors are obtained from the fluctuation-dissipation relation,

$$S_{\frac{D}{S}}\left(q,\omega \right)=-\frac{1}{\pi }\;\left[\frac{1}{1-e^{-\frac{\omega }{T}}}\right]\text{Im}\;\chi_{\frac{D}{S}}\left(q,i\omega_{n}\;\to \;\omega +i\delta \right)\text{,}$$

where q and ω are the transferred momentum and energy, respectively, and δ = 0.003 is the numerical broadening used in the analytic continuation. The spectra are evaluated along the high-symmetry path [0,0] → [π,0] → [π,π] → [0,π] → [0,0] of the first Brillouin zone and, where required, along additional cuts designed to resolve the low-q collective modes and the roton feature around [π,π].

#### Collective modes and roton tracking

The main collective-mode branches are identified from the poles of the RPA response function, equivalently from the zeros of $\det\left[I-\chi 0\left(q,i\omega_{n}\right)UM_{I}\right]$. Following our earlier analysis, the dominant branch is tracked from the condition $Re\Sigma =0\;with\;\Sigma =\chi_{34}^{0}\chi_{43}^{0}-{\left(\chi_{12}^{0}\right)}^{2}$. The sound speed in the density channel and the bogolon speed in the spin channel are extracted from linear fits to the low-momentum peak positions, c = ω/q. To determine the Cooper-pair momentum independently from the excitation spectrum, we track the displacement of the roton minimum away from [π,π] in the half-filled system and compare it with the value of Q obtained from the self-consistent mean-field solution. This comparison forms the roton-based protocol proposed in the manuscript for measuring the center-of-mass momentum of Cooper pairs.

#### Numerical implementation

All calculations were performed using self-written Fortran codes. Momentum-space summations were evaluated on a discrete k-space grid covering the first Brillouin zone. Self-consistency was reached when the changes in μ, Δ, and Q were smaller than $10^{-15}$. Figures were prepared using origin2018.

### Quantification and statistical analysis

This study does not involve statistical hypothesis testing or biological replicate-based inference.

## Supplemental information

Document S1. Method S1
