# Supplementary material for: Novel dynamics and Cooper-pair momentum measurement in Fulde-Ferrell-Larkin-Ovchinnikov superfluids on optical lattices
Source: iScience. 2026 May 26;29(6):116065. doi: 10.1016/j.isci.2026.116065 (PMC13233767; doi:10.1016/j.isci.2026.116065)
Supplement: Document S1. Method S1 [file mmc1.pdf]

iScience, Volume *29*

Supplemental information

Novel dynamics and Cooper-pair momentum measurement in Fulde-Ferrell-Larkin-Ovchinnikov superfluids on optical lattices

Shuning Tan, Jiayi Shi, Peng Zou, Tianxing Ma, and Huaisong Zhao

Supplemental information

Supplemental analytical expressions for the RPA response functions

Analytical forms of the response functions

The mean-field response function χ^0 of 2D FFLO Fermi superfluid on an optical lattice is numerically calculated, and all 9 independent matrices elements of χ^0 are displayed as,

$$\begin{aligned}
\chi^0_{11} &= \sum_{\mathbf{k}} U^2_{\mathbf{k+q}}[U^2_{\mathbf{k}}L_1(\mathbf{k},\mathbf{q},i\omega_n) + V^2_{\mathbf{k}}L_2(\mathbf{k},\mathbf{q},i\omega_n)] \\
&- \sum_{\mathbf{k}} V^2_{\mathbf{k+q}}[U^2_{\mathbf{k}}L_3(\mathbf{k},\mathbf{q},i\omega_n) - V^2_{\mathbf{k}}L_4(\mathbf{k},\mathbf{q},i\omega_n)],
\end{aligned}$$

$$\begin{aligned}
\chi^0_{12} &= -\sum_{\mathbf{k}} \frac{\Delta_0^2}{4E_{\mathbf{k}}E_{\mathbf{k+q}}}[L_5(\mathbf{k},\mathbf{q},i\omega_n) - L_6(\mathbf{k},\mathbf{q},i\omega_n)] \\
&- \sum_{\mathbf{k}} \frac{\Delta_0^2}{4E_{\mathbf{k}}E_{\mathbf{k+q}}}[L_7(\mathbf{k},\mathbf{q},i\omega_n) + L_8(\mathbf{k},\mathbf{q},i\omega_n)],
\end{aligned}$$

$$\begin{aligned}
\chi^0_{13} &= \sum_{\mathbf{k}} \frac{\Delta_0}{2E_{\mathbf{k+q}}}[U^2_{\mathbf{k}}L_1(\mathbf{k},\mathbf{q},i\omega_n) + V^2_{\mathbf{k}}L_2(\mathbf{k},\mathbf{q},i\omega_n)] \\
&+ \sum_{\mathbf{k}} \frac{\Delta_0}{2E_{\mathbf{k+q}}}[U^2_{\mathbf{k}}L_3(\mathbf{k},\mathbf{q},i\omega_n) - V^2_{\mathbf{k}}L_4(\mathbf{k},\mathbf{q},i\omega_n)],
\end{aligned}$$

$$\begin{aligned}
\chi^0_{14} &= \sum_{\mathbf{k}} \frac{\Delta_0}{2E_{\mathbf{k}}}U^2_{\mathbf{k+q}}[L_1(\mathbf{k},\mathbf{q},i\omega_n) - L_2(\mathbf{k},\mathbf{q},i\omega_n)] \\
&- \sum_{\mathbf{k}} \frac{\Delta_0}{2E_{\mathbf{k}}}V^2_{\mathbf{k+q}}[L_3(\mathbf{k},\mathbf{q},i\omega_n) + L_4(\mathbf{k},\mathbf{q},i\omega_n],
\end{aligned}$$

$$\begin{aligned}
\chi^0_{22} &= \sum_{\mathbf{k}} V^2_{\mathbf{k}}[V^2_{\mathbf{k+q}}L_5(\mathbf{k},\mathbf{q},i\omega_n) + U^2_{\mathbf{k+q}}L_6(\mathbf{k},\mathbf{q},i\omega_n)] \\
&- \sum_{\mathbf{k}} U^2_{\mathbf{k}}[V^2_{\mathbf{k+q}}L_7(\mathbf{k},\mathbf{q},i\omega_n) - U^2_{\mathbf{k+q}}L_8(\mathbf{k},\mathbf{q},i\omega_n)],
\end{aligned}$$

$$\begin{aligned}
\chi^0_{23} &= -\sum_{\mathbf{k}} \frac{\Delta_0}{2E_{\mathbf{k+q}}}V^2_{\mathbf{k}}[L_5(\mathbf{k},\mathbf{q},i\omega_n) - L_6(\mathbf{k},\mathbf{q},i\omega_n)] \\
&+ \sum_{\mathbf{k}} \frac{\Delta_0}{2E_{\mathbf{k+q}}}U^2_{\mathbf{k}}[L_7(\mathbf{k},\mathbf{q},i\omega_n) + L_8(\mathbf{k},\mathbf{q},i\omega_n)],
\end{aligned}$$

$$\begin{aligned}
\chi^0_{24} &= -\sum_{\mathbf{k}} \frac{\Delta_0}{2E_{\mathbf{k}}}[V^2_{\mathbf{k+q}}L_5(\mathbf{k},\mathbf{q},i\omega_n) + U^2_{\mathbf{k+q}}L_6(\mathbf{k},\mathbf{q},i\omega_n)] \\
&- \sum_{\mathbf{k}} \frac{\Delta_0}{2E_{\mathbf{k}}}[V^2_{\mathbf{k+q}}L_7(\mathbf{k},\mathbf{q},i\omega_n) - U^2_{\mathbf{k+q}}L_8(\mathbf{k},\mathbf{q},i\omega_n)],
\end{aligned}$$

$$\begin{aligned}\chi^0_{34} &= \sum_{\mathbf{k}} U^2_{\mathbf{k+q}}[V^2_{\mathbf{k}} L_1(\mathbf{k},\mathbf{q},i\omega_n) + U^2_{\mathbf{k}} L_2(\mathbf{k},\mathbf{q},i\omega_n)] \\ &- \sum_{\mathbf{k}} V^2_{\mathbf{k+q}}[V^2_{\mathbf{k}} L_3(\mathbf{k},\mathbf{q},i\omega_n) - U^2_{\mathbf{k}} L_4(\mathbf{k},\mathbf{q},i\omega_n)], \\ \chi^0_{43} &= \sum_{\mathbf{k}} V^2_{\mathbf{k}}[U^2_{\mathbf{k+q}} L_5(\mathbf{k},\mathbf{q},i\omega_n) + V^2_{\mathbf{k+q}} L_6(\mathbf{k},\mathbf{q},i\omega_n)] \\ &- \sum_{\mathbf{k}} U^2_{\mathbf{k}}[U^2_{\mathbf{k+q}} L_7(\mathbf{k},\mathbf{q},i\omega_n) - V^2_{\mathbf{k+q}} L_8(\mathbf{k},\mathbf{q},i\omega_n)]. \end{aligned} \tag{S1}$$

Kernel functions

The corresponding functions $L_1(\mathbf{k},\mathbf{q},i\omega_n)$, $L_2(\mathbf{k},\mathbf{q},i\omega_n)$, $L_3(\mathbf{k},\mathbf{q},i\omega_n)$, $L_4(\mathbf{k},\mathbf{q},i\omega_n)$, $L_5(\mathbf{k},\mathbf{q},i\omega_n)$, $L_6(\mathbf{k},\mathbf{q},i\omega_n)$, $L_7(\mathbf{k},\mathbf{q},i\omega_n)$, and $L_8(\mathbf{k},\mathbf{q},i\omega_n)$ are shown as

$$\begin{aligned} L_1(\mathbf{k},\mathbf{q},i\omega_n) &= \frac{n_F(E^{(1)}_{\mathbf{k}}) - n_F(E^{(1)}_{\mathbf{k+q}})}{i\omega_n + E^{(1)}_{\mathbf{k}} - E^{(1)}_{\mathbf{k+q}}} \\ L_2(\mathbf{k},\mathbf{q},i\omega_n) &= \frac{1 - n_F(E^{(2)}_{\mathbf{k}}) - n_F(E^{(1)}_{\mathbf{k+q}})}{i\omega_n - E^{(2)}_{\mathbf{k}} - E^{(1)}_{\mathbf{k+q}}} \\ L_3(\mathbf{k},\mathbf{q},i\omega_n) &= \frac{1 - n_F(E^{(1)}_{\mathbf{k}}) - n_F(E^{(2)}_{\mathbf{k+q}})}{i\omega_n + E^{(1)}_{\mathbf{k}} + E^{(2)}_{\mathbf{k+q}}} \\ L_4(\mathbf{k},\mathbf{q},i\omega_n) &= \frac{n_F(E^{(2)}_{\mathbf{k+q}}) - n_F(E^{(2)}_{\mathbf{k}})}{i\omega_n - E^{(2)}_{\mathbf{k}} + E^{(2)}_{\mathbf{k+q}}} \\ L_5(\mathbf{k},\mathbf{q},i\omega_n) &= \frac{n_F(E^{(1)}_{\mathbf{Q-k-q}}) - n_F(E^{(1)}_{\mathbf{Q-k}})}{i\omega_n - E^{(1)}_{\mathbf{Q-k}} + E^{(1)}_{\mathbf{Q-k-q}}} \\ L_6(\mathbf{k},\mathbf{q},i\omega_n) &= \frac{1 - n_F(E^{(2)}_{\mathbf{Q-k-q}}) - n_F(E^{(1)}_{\mathbf{Q-k}})}{i\omega_n - E^{(1)}_{\mathbf{Q-k}} - E^{(2)}_{\mathbf{Q-k-q}}} \\ L_7(\mathbf{k},\mathbf{q},i\omega_n) &= \frac{1 - n_F(E^{(1)}_{\mathbf{Q-k-q}}) - n_F(E^{(2)}_{\mathbf{Q-k}})}{i\omega_n + E^{(2)}_{\mathbf{Q-k}} + E^{(1)}_{\mathbf{Q-k-q}}} \\ L_8(\mathbf{k},\mathbf{q},i\omega_n) &= \frac{n_F(E^{(2)}_{\mathbf{Q-k}}) - n_F(E^{(2)}_{\mathbf{Q-k-q}})}{i\omega_n + E^{(2)}_{\mathbf{Q-k}} - E^{(2)}_{\mathbf{Q-k-q}}}, \end{aligned} \tag{S2}$$

where the function $n_F(x)$ is the Fermi distribution.
